# Application of Raw and Defatted by Supercritical CO_2_ Hemp Seed Press-Cake and Sweet Grass Antioxidant Extract in Pork Burger Patties

**DOI:** 10.3390/foods10081904

**Published:** 2021-08-16

**Authors:** Kristi Kerner, Ivi Jõudu, Alo Tänavots, Petras Rimantas Venskutonis

**Affiliations:** 1 Chair of Food Science and Technology, Institute of Veterinary Medicine and Animal Sciences, Estonian University of Life Sciences, Fr. R. Kreutzwaldi 56/5, 51006 Tartu, Estonia; kristi.kerner@emu.ee (K.K.); ivi.joudu@emu.ee (I.J.); 2 Department of Food Science and Technology, Kaunas University of Technology, Radvilenu pl. 19, LT-50254 Kaunas, Lithuania; 3 ERA Chair for Food (By-) Products Valorisation Technologies (VALORTECH), Estonian University of Life Sciences, Fr. R. Kreutzwaldi 56/5, 51006 Tartu, Estonia; 4 Chair of Animal Breeding and Biotechnology, Institute of Veterinary Medicine and Animal Sciences, Estonian University of Life Sciences, Fr. R. Kreutzwaldi 62, 51006 Tartu, Estonia

**Keywords:** pork burgers, oxidation, sweet grass antioxidant, hemp seed press-cake

## Abstract

There is an increasing tendency toward the application of plant origin ingredients in meat products. This study evaluates the physicochemical properties and oxidative stability of pork burger patties produced with the addition of dried raw and defatted by supercritical CO_2_ extraction hemp seed press-cake as protein-rich ingredients (1.5–2.0%) and sweet grass ethanolic extract (0.5%) as a strong natural antioxidant. The main aim of using such a combination was to assess the possibility of mitigating the negative effects of hemp seed press-cake, containing approx. 10% of highly unsaturated oil, on the oxidation of meat products. The patties were compared with the control sample (without additives) during storage on days 0, 4, 8, 15, and 21 at 4 °C in modified atmosphere conditions. Plant ingredients reduced the lightness of pork patties, while their effects on other physicochemical characteristics were insignificant. The patties with fully defatted hemp seed flour showed the lowest grilling losses. Based on the measurement of thiobarbituric acid reactive substances, raw hemp seed press-cake increased the oxidative rate of pork patties; however, remarkably, the addition of sweet grass extract completely inhibited oxidation during the whole period of storage. The sensory characteristics of the products were acceptable; however, the patties with sweet grass extract received lower evaluation scores.

## 1. Introduction

Various (bio)chemical reactions and microbiological processes occur in the meat during processing and storage; some of them may adversely affect the quality of raw and processed products. The oxidation of the main meat constituents such as lipids and proteins is among the most important factors of deterioration. The undesirable changes in meat products can be controlled by various physical methods and chemical additives. Due to increasing consumer preferences towards food ’naturalness’, there is a tendency to replace chemical preservatives with natural plant origin alternatives. Numerous plant materials are a good source of natural antioxidants and antimicrobial agents [1], which help to extend product shelf-lives by stabilising their quality characteristics. Moreover, natural ingredients may increase the nutritional value and health benefits of meat products by enriching them with bioactive phytochemicals and other valuable nutrients, such as polyphenolic compounds, vitamins, dietary fibres, and minerals. Another important tendency in the development of meat products is related to the use of cheaper plant-origin protein substitutes in their formula [2]. Following this tendency, various plant origin proteinaceous ingredients have been tested in meat products, including the most widely tested and used preparations from soya, pea, mung bean, rice, and lupin [2,3,4]. Other, so-called ‘forgotten’ and emerging crops have also attracted increasing interest both for researchers and the industry. From this point of view, industrial hemp (*Cannabis sativa* L.) has become one of the most popular plants in the last decade after favourable regulatory changes for its wider cultivation [5].

Hemp inflorescences biosynthesise quite unique health beneficial phytocannabinoids, while hemp seeds are an excellent source of high-value oil, proteins, and valuable micronutrients [6], including health beneficial bioactive compounds [7,8]. For instance, Pihlanto et al. [9] reported 5.88–10.63 mg total phenolic content (expressed in mg of gallic acid equivalents) per gram of dry defatted hemp seed flour, which was also confirmed by Rea et al. [10]. However, the reports on the use of hemp ingredients in meat products are still rather scarce. For instance, most recently, Zahari et al. [11] developed meat substitutes with hemp protein using extrusion cooking. However, mechanically pressed hemp seed press-cakes with 35–45% of proteins still contain approximately 10% of highly unsaturated and are very sensitive to oxidation residual oil. Therefore, the direct addition of such a press-cake into meat products may negatively affect their quality. Consequently, before using hemp seed press-cake in meat products, the residual oil should be removed by using more efficient extraction methods, while in the case using non-defatted press-cake, the use of antioxidants may be required to mitigate the possible adverse effects of the residual unsaturated oil.

The hypothesis of the current work is based on the following assumptions: (1) mechanically pressed and dried hemp seed flour ingredients may have inferior effects on meat product quality due to the oxidation of residual highly unsaturated hemp oil, while the removal of residual oil with supercritical CO_2_ extraction should eliminate oxidation-related negative effects; (2) a strong natural antioxidant extracted from sweet grass may inhibit the oxidation of meat products produced with non-defatted hemp seed flour and may therefore mitigate its possible negative effects. In order to test these hypotheses, hemp seed press-cake products as protein-rich additives and sweet grass ethanolic extract, containing a strong radical scavenger 5,8-dihydroxycoumarin and demonstrating powerful antioxidant potential [12,13,14], were tested in the pork meat burger patties.

## 2. Materials and Methods

### 2.1. Chemicals and Reagents

2,2′-Azino-bis(3-ethylbenzthiazoline-6-sulphonic acid), (ABTS); 2,2-diphenyl-1-picrylhydrazyl hydrate free radical (DPPH^•^, 95%); gallic acid (GA, 3,4,5-trihydroxybenzoic acid, 99%); 6-hydroxy-2,5,7,8-tetramethylchroman-2-carboxylic acid (Trolox, 97%); and Na_2_CO_3_ were purchased from Sigma-Aldrich (Steinheim, Germany). Folin–Ciocalteu’s phenol reagent (2 M); 2-(3-hydroxy-6-oxo-xanthen-9-yl)benzoic acid (fluorescein); and 2,2′-azobis-2-methyl-propanimidamide dihydrochloride (AAPH) were from Fluka Analytical (Bornem, Belgium). KCl, NaCl, K_2_S_2_O_8_, and KH_2_PO_4_ were from Lach-Ner (Brno, Czech Republic). Na_2_HPO_4_ and isoamyl alcohol (a mixture of isomers) were from Merck KGaA (Darmstadt, Germany). Agricultural origin ethanol (96.3%) was from Stumbras (Kaunas, Lithuania). Liquid nitrogen was from AGA SIA (Riga, Latvia). CO_2_ and N_2_ gases (99.9%) were from Gaschema (Jonava region, Lithuania). Perchloric acid; 2-thiobarbituric acid; 1,1,3,3 tetraethoxypropane; butylated hydroxytoluene; sulfuric acid; and boric acid were purchased from Sigma-Aldrich Chemie (Steinheim, Germany). Sodium hydroxide (NaOH, 50%) was from Ingle AS (Ingliste, Estonia), and Kjeltabs FOSS Analytical A/S was from Oridor Eesti OÜ (Tartu, Estonia).

### 2.2. Plant Ingredients for the Production of Pork Burger Patties

Dried and mechanically pressed hemp seed cake, containing 36.6 g/100 g of protein, 13.3 g/100 g of fat, and 21.0 g/100 g dietary fibre was kindly donated by the company Agropro (Kaunas, Lithuania). Part of the press-cake batch was defatted by supercritical CO_2_ extraction (SFE-CO_2_) in a pilot 10 L extractor (Applied Separations, Allentown, PA, USA) at 350 MPa pressure and 60 °C temperature for 4 h, when the extraction kinetics curve reached the plateau. Dried sweet grass (*Hierochloe odorata*) was purchased from the company Mėta (Vaidotai, Vilnius, Lithuania). Previously optimised for herbal materials, high-pressure extraction procedures were applied to sweet grass with slight modifications [15]. First, the herb was ground and extracted in a pilot-scale extractor with supercritical CO_2_ at 40 MPa and 60 °C for removing lipophilic substances and volatile aroma constituents (sweet grass possesses a strong specific aroma of coumarin). Afterwards, the defatted and deodorised material was extracted with ethanol in an accelerated solvent extractor ASE 350 (Dionex, Sunnyvale, CA, USA) at 10.3 MPa and 100 °C, using 3 cycles, 15 min each. The solvent was removed in a Buchi rotary vacuum evaporator (Flawil, Switzerland) at 40 °C.

### 2.3. Determination of Antioxidant Properties of Sweet Grass Extract

The total phenolic content was measured with Folin–Ciocalteu reagent as originally described by Singleton and Orthofer [16]. In brief, 30 μL of a sample (0.1%) was mixed with 150 μL of 10-fold diluted in distilled water Folin–Ciocalteu reagent and 120 μL of 7.5% Na_2_CO_3_ in the microplate wells, and after shaking for 30 s and incubating for 30 min at room temperature, the absorbance was measured at 765 nm. A series of GA solutions in the concentration range of 0.025–0.350 mg/mL was used for the calibration curve (regression equation: absorbance = 10.895 × GA conc. + 0.0729). The results were expressed in mg of GA equivalents per g of dry extract weight (mg GAE/g dw).

The DPPH^•^ scavenging capacity (RSC) of extracts was determined by a slightly modified method of Brand-Williams et al. [17] using a 96-well microplate reader FLUOstar Omega (BMG Labtech, Offenburg, Germany). In brief, 7.5 μL of extract was mixed with 300 μL DPPH^•^ solution and the decrease in absorbance was measured at 515 nm. The RSC values were calculated using a regression equation: absorbance = 340.62 × Trolox conc. + 7.8965 (R^2^ = 0.99) produced with different concentrations of synthetic antioxidant Trolox. The RSC is expressed in milligrams of Trolox equivalent (TE)/g dw. In addition, an effective DPPH^•^·inhibitory concentration (IC_50_) was determined graphically.

An ABTS^•+^ decolourisation assay was performed following a slightly modified method of Re et al., 1999. In brief, 6 µL of the sample was added to 294 µL of ABTS^•+^ working solution, which was prepared by mixing 50 mL of ABTS (2 mM) with 200 µL of K_2_S_2_O_8_ (70 mM) and by keeping it in the dark for 15–16 h before use. The working solution was prepared by diluting with PBS (8.18 g NaCl, 0.27 g KH_2_PO_4_, 1.78 g Na_2_HPO_4_ × 2 H_2_O, and 0.15 g KCl in 1 L of distilled H_2_O) to obtain the absorbance of 0.800 ± 0.030 at 734 nm. The absorbance was measured in a 96-well microplate using a FLUOstar Omega Reader during 30 min at 734 nm. A series of Trolox solutions (399–1198 μM/L) were used for calibration. The results are expressed as µM TE/g dw.

An ORAC (Oxygen Radical Absorbance Capacity) assay was performed using fluorescein as a fluorescent probe and AAPH as a peroxyl radical generator [18]. In brief, 25 µL of the sample was pipetted into 150 µL (14 μM) fluorescein solution and, after incubating for 15 min at 37 °C, 25 µL of AAPH (240 mM) was added. The fluorescence was recorded in a FLUOstar Omega Reader every cycle (in total, 120 cycles) using 485 excitation and 530 emission fluorescence filters. Antioxidant curves (fluorescence versus time) were first normalised, and from the normalised curves, the net area under the fluorescein decay curve (AUC) was calculated: AUC = (1 + f_1_/f_0_ + f_2_/f_0_… f_i/_f_0_) × CT, where f_0_ is the initial fluorescence reading at 0 min, f_i_ is the fluorescence reading at time I, and CT is cycle time in min. The final ORAC values were calculated using a regression equation between Trolox concentration and the net AUC. Trolox solutions (0–250 μM) were used for the calibration. The results were expressed as µM TE/g dw.

All antioxidant measurements were carried out in six replicates.

### 2.4. Preparation of Pork Burger Patties

Minced pork meat (moisture 67.82%, protein 18.62%, fat 12.25%, and ash 0.98%) was purchased from a local commercial abattoir, and salt and black pepper were purchased from a local food store (Tartu, Estonia). The mixture was prepared with tap water, salt, and black pepper and mixed manually until all of the ingredients were spread evenly. The batter was divided into five portions: (1) control samples (83.5% of minced pork meat, 15% water, 1.5% salt, and 0.2% black pepper); (2) samples with 2% dried hemp seed press-cake flour (RH); (3) samples with 2% fully defatted hemp seed press-cake flour (DH); (4) samples with 0.5% sweet grass extract (SG); and (5) samples with SG and RH, 0.5 and 1.5%, respectively (RHSG). The raw mixture was pressed into 70 g patties (Ø 8.6 cm) using a hamburger press (Indasia, Greece). The patties were cooked in a preheated teflon-coated grill Sage Smart Grill Pro Model BGR840 BSS (Breville, Sydney, Australia) to an internal temperature of 75 °C measured with temperature probe of the grill. The patties were cooled down to room temperature and packed by using modified atmosphere consisting of 70% N_2_ and 30% CO_2_ (Linde GAS AS, Tallinn, Estonia) with a Vision Pack Srl VP01 (Packaging Factory Holding, Lallio, Italy). All samples were stored in cooled condition at 4 °C. Tests were conducted at 0, 4, 8, 15, and 21 days of storage.

### 2.5. Determination of Quality Characteristics

Grilling loss was measured after cooling the cooked products to room temperature by weighing the patties before and after the thermal treatment. The samples for the chemical analyses were ground and homogenised in a Retsch GM200 laboratory homogeniser (Retsch Gmbh & Co, Haan, Germany). The cooked patties were analysed for moisture (EVS-ISO 1442:1999), protein (EVS-ISO 937:1978, Kjeldahl method), fat (EVS-ISO 2446:2001, Gerber method), and ash content (ISO 936:1999).

For pH, 5 g of sample was homogenised with 50 mL of 0.1 M potassium chloride solution in Retsch GM200 (ISO 2917:1999) and measured with a Seven 2Go™ pH-meter (Mettler-Toledo AG Analytical, Schwerzenbach, Switzerland). A pH meter was calibrated with pH 4 and pH 7 buffer solutions at room temperature. Water activity (aw) was determined in a water activity analyser (Aqua Lab, Model Series 3 TE, Decagon Devices, Inc., Washington, DC, USA) by placing the sample in a tightly closed chamber, where the air was humidified or dehumidified to achieve equilibrium humidity.

The colour was measured using a X-Rite 964 spectrophotometer (X-Rite, Grand Rapids, MI, USA) and expressed by CIE (International Commission on Illumination) Lab system values (D65 and observer angle of 10°), namely L*—lightness, a*—redness, and b*—yellowness. The colorimeter was calibrated on a black and white surface by the manufacturer’s specifications. The patties were cut into halves immediately after opening the package, and three replicate measurements were taken on the internal area of the freshly cut surface.

The total colour difference (Δ*E_Lab_*) calculation between the control and test samples was based on the three colour coordinates CIE L*, a*, and b* (Equation (1)).

(1) $$∆E_{Lab}=\sqrt{{\left(L_{0}^{*}-L_{1}^{*}\right)}^{2}+{\left(a_{0}^{*}-a_{1}^{*}\right)}^{2}+{\left(b_{0}^{*}-b_{1}^{*}\right)}^{2},}$$

where $∆E_{Lab}$ is the total colour difference between the control and test samples; $\;L_{0}^{*},\;a_{0}^{*},\;and\;b_{0}^{*}$ are the means of the colour parameters determined for the control samples; and $L_{1}^{*},\;a_{1}^{*},\;and\;b_{1}^{*}$ are the means of the colour parameters determined for the test samples.

In the interpretation of the results, the following was assumed:

- when 0 < ∆*E_Lab_* < 1—the observer does not notice the difference;
- when 1 < ∆*E_Lab_* < 2—only an experienced observer may notice the difference;
- when 2 < ∆*E_Lab_* < 3.5—an unexperienced observer also notices the difference;
- when 3.5 < ∆*E_Lab_* < 5—a clear difference in colour is noticed and;
- when 5 < ∆*E_Lab_*—an observer notices two different colours [19].

The formation of the oxidation products was evaluated by measuring the thiobarbituric acid reactive substances (TBARS) as reported by Pikul et al. [20] with some modifications. Five grams of the sample were homogenised with 20 mL of 4% perchloric acid and 0.25 mL of butylated hydroxytoluene in an Ultra-Turax IKA T18 homogeniser (IKA, Staufen, Germany) and filtered. The filtrate with TBA was heated in a water bath at 80 °C 1 h and cooled. The absorbance was determined at 538 nm; 1,1,3,3 tetraethoxypropane was used for calibration curve. TBARS were measured at the timed periods, and expressed in malondialdehyde (MDA) mg/kg, while the changes are also expressed in MDA mg/kg by subtracting the measured value during storage from the value measured on day 0 (ΔMDA mg/kg).

### 2.6. Assessment of Sensory Attributes

The sensory assessment of raw and grilled pork patties was carried out by eight randomly selected experienced assessors (previously completed sensory training) from the Estonian University of Life Sciences, Chair of Food Science and Technology, to obtain a more professional evaluation for initial assessment. They were instructed with the evaluation procedure. Special evaluation sheets were worked out to describe the sensory characteristics of the samples. The sensory analysis was carried out in a room with individual booths. Stored grilled patties were warmed to 60 °C in a microwave oven (Moulinex Micro-Chef V98, Ecully, France) and then halved before the evaluation. The grilled patties were assessed on days 0, 4 and 8, and raw patties were assessed on days 0 and 8. The samples of raw and grilled patties were pre-coded and presented to the assessors on white dishes. Water was provided between the samples of grilled patties.

The attributes for the evaluation of grilled patties were appearance, colour, odour, taste, texture, and juiciness. The appearance, colour, and odour grades were recorded for raw patties. A hedonic 9-point scale (9—very good, 5—satisfying, and 1—not satisfying) was used for sensory evaluation as this approach has been quite widely used for comparison purposes, particularly in cases using new ingredients [21].

### 2.7. Statistical Data Analysis

Statistical analyses were performed with the statistical package R 4.0.4 [22]. The effects of variants, storage period, and their interaction and the random effect of four batches (experimental replications) on the samples’ pH, colour characteristics, a_w_, and TBARS were studied by the Linear Mixed-Effects Model (GLMM). The Emmeans [23] and multcomp [24] packages were used to carry out the pairwise comparison of the groups. Tukey’s multiple comparison post hoc test was used to determine the groups’ least square mean differences at the significance level of α = 0.05. The effects of variants and four batches on the sample moisture, protein, and ash content as well as on the grill loss were measured only on day 0 by GLMM. All model-assessed results are presented as least-square means. Boxplots charts were used to illustrate the results of the sensory evaluation by the ggplot2 [25] package in R 4.0.4 [22].

## 3. Results and Discussion

### 3.1. Characterisation of Ingredients

The preparation of plant ingredients for traditional meat products is an important process, which should consider various requirements. In the case using extractives, preferably environment and food-friendly (green) solvents and methods should be used, while the sensory characteristics of new ingredients should be acceptable for the products. In this study, modern and green extraction methods are used both for the removal of lipophilic compounds and obtaining protein-enriched defatted hemp press-cake and strong sweet grass antioxidant extract. In addition, SFE-CO_2_ removes volatile compounds and, therefore, the odour of the ingredients obtained becomes very weak. Non-defatted hemp press-cake possesses a strong nutty odour, while sweet grass has a strong coumarin-like aroma, which is not desired in meat products. In addition, the levels of tolerable daily intake (TDI) of coumarin are restricted to 0.1 mg/kg body weight [26]. Hemp seeds and their mechanically produced press-cake may contain some micro-residues of psychotropic tetrahydrocannabinol (THC), which is also fairly soluble in supercritical CO_2_ and is removed during SFE-CO_2_ [27,28].

Consequently, the applied methods produce innovative and free from hazardous compounds ingredients. Thus, defatted hempseed press-cake contained 51.7 g/100 g of protein, 1.4 g/100 g of fat, and 26.1 g/100 g of dietary fibre; it had a weak odour, while its colour was remarkably lighter compared with the non-defatted press-cake. Sweet grass extract was a very strong antioxidant (Table 1), particularly in ORAC assay, which is, among more widely used in vitro chemical assays, more relevant to the oxidation events occurring in biosystems [18,29]. Sweet grass extracts were reported as being very strong antioxidants in rapeseed oil [30], and later 5,8-dihydroxycoumarin and its glycoside were identified as the main radical scavengers and new natural compounds [12]. Recently, Martinez et al. [8] reported that the fractions isolated with a hydroethanolic mixture and ethyl acetate from the defatted press-cake with hexane hempseeds contained N-*trans*-caffeoyltyramine as one of the main bioactives and reduced the inflammatory competence of lipopolysaccharide-treated human primary monocytes.

### 3.2. Proximate Composition and Grilling Losses of Pork Burger Patties

As was expected, the addition of a not very high amount of plant origin additives did not have any remarkable effect on the content of the main components in grilled meat burgers (Table 2). For instance, there were no significant effects on the content of proteins and minerals. However, ANOVA indicated a significantly lower amount of moisture in the samples with RH and SG and a significantly higher amount of fat in the product with SG. Grilling loss affects the juiciness of patties, which is linked to consumer preferences and the production profitability, and were significantly lower in the samples with DH. Due to the compositional complexity of meat products as well as the multifunctional effects of grilling, it is rather difficult to explain the indicated differences. In addition, it should also be noted that the SDs were rather high for all measured characteristics, which is natural for experiments with highly heterogeneous biomaterials. The lowest grilling loss was in DH (*p* < 0.05), most likely explained by the high protein content in this ingredient, which may strongly absorb the water present in raw meat. The same tendency may be observed in the samples with RH, while in the case using RH with SG, the effect of hemp seed press-cake may be explained by its water-binding capacity. Raikos et al. [31], Xu et al. [6], and Zając et al. [32] reported that hemp protein or hemp flour with high protein content improves the products’ water holding capacity. Therefore, the lower grilling losses with hemp additives may be useful for producers to help retain the moisture inside the product.

A higher amount of fat determined in the samples with SG may not be explained straightforwardly by the addition of 0.5% of the lipid-free plant extract. Most likely, the increase was determined due to the higher grilling losses and lower moisture in the SG samples causing the proportional increase in fat content. However, it is interesting to note that, in the case using SG together with RH, the grilling losses were significantly lower than in the control sample and the product with the separately applied SG. The reason is not clear; however, it may be preliminary hypothesised that polar antioxidants in SG, in this case, interact with hemp proteins, and therefore interfere with water polar molecules and provide some effects on the overall grilling losses in the complex meat system. In general, it is evident that the effects of used plant additives were not important for proximate composition of the grilled meat products.

### 3.3. Changes of pH and Water Activity (a_w_) during Storage of Pork Patties

The pH of meat and meat products is a quality parameter related to its safety, technological and sensory properties [33,34,35]. The measured pH values of the grilled patties ranged within 6.1–6.3 (Table 3). It is evident that hemp additives slightly, although in most cases significantly, increased a meat product’s pH after grilling and during the whole period of storage, while SG has no effects on this characteristic. A significantly higher value of pH of burgers with hemp seed additives may be due to the addition of a small amount of buffer-type compounds present in hemp [36,37,38]. It is highly unlikely that the fluctuations in pH values within the measured range have any noticeable changes on the other quality characteristics of meat products. Some very small, but significant (*p* < 0.05) increases in pH were observed in the samples with RH after 15 and 21 days of storage; it may be explained by the formation of acidic oxidation products of residual hemp oil.

The water activity (aw) of meat products is usually sufficiently high for various microbiological and (bio)chemical processes. Consequently, it is an important factor in terms of product stability during storage. The aw values of all pork patties during the whole period of storage were in the range of 0.950–0.963 (Table 4). Again, only slight differences were determined for the samples prepared with hemp press-cake and sweet grass extract, although ANOVA indicated significantly higher values for almost all stored samples with additives compared with the control.

### 3.4. Effect of Additives on Colour

Colour is an important quality parameter of meat products for consumers in terms of their purchasing preferences [33,37,39]. In addition, colour characteristics are related to several important processes occurring in meat during processing and storage. Plant-based ingredients may have strong effects on the colour of meat products. The effect of different plant-based ingredients on colour parameters (L*, a*, and b*) has been evaluated by several researchers [33,37,40,41,42], who investigated how the ingredients enhancing meat nutritional quality influence their overall acceptance and appearance in the eyes of the consumers.

All additives in the current study had different green colour taints and intensity, most likely due to the presence and composition of chlorophylls. For instance, SG possessed a dark green colour, RH had a lighter green colour, while the green colour of DH (the lightest ingredient) was less evident. Chlorophylls are soluble in supercritical CO_2_, and most of them are removed during the extraction of residual lipids. It is evident that all additives significantly decreased the L* value (Table 5); however, the effect of dark green SG was remarkably stronger than that of DH and RH, which agrees with the visual colour appearance of these ingredients. It is interesting to note that significant changes in L* during storage (some increase) were observed only for the samples with SG, most likely, due to the degradation of added with SG chlorophylls and the effects of antioxidants on meat pigments. This additive also significantly increased the product yellowness (b*). Some changes in b* have been observed during the storage of meat patties with SG and RHSG. The effects of the additives on the redness (a*) was significant neither in the freshly grilled products nor in the stored ones: this important meat colour attribute remained stable during the whole storage period.

**Figure 1:**
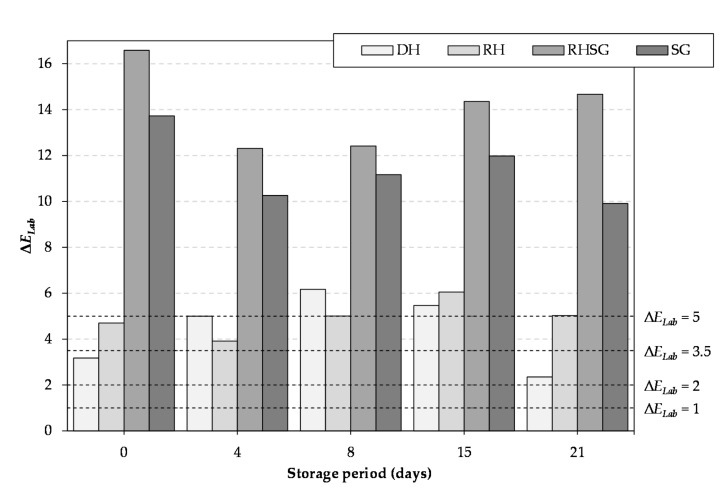
The total colour difference (Δ*E_Lab_*) between control and test samples during the storage period: 0 < ∆*E_Lab_* < 1—the observer does not notice a difference, 1 < ∆ *E_Lab_* < 2—only an experienced observer may notice the difference, 2 < ∆*E_Lab_* < 3.5—an unexperienced observer also notices the difference, 3.5 < ∆*E_Lab_* < 5—a clear difference in colour is noticed, and 5 < ∆*E_Lab_*—an observer notices two different colours (RH—with 2% dried mechanically pressed hempseed cake, DH—with 2% defatted by supercritical CO_2_ extraction hempseed cake, RHSG—with 0.5% sweetgrass extract and 1.5% dried pressed hemp seed-cake, and SG—0.5% sweet grass extract).

All of the used additives affected the total colour difference (Δ*E_Lab_*) between the control and test samples during storage period (Figure 1). According to the results, even an unexperienced observer can notice the difference in colour (∆*E_Lab_* > 2) between the control sample and samples with the additives, especially regarding samples with RHSG and SG (Δ*E_Lab_* > 5), which may be mainly due to the presence of dark green colour of sweet grass. However, being a lighter green, DH and RH had clear effects on the colour difference (∆*E_Lab_* > 2) compared with the control sample. This indicates that there is necessity to find a method to decrease the colour-changing effect of the additives in the case of development of a consumer-ready product.

### 3.5. The Effect of Ingredients on the Formation of Oxidation Products (TBARS) in Pork Patties during the Storage

The degradation products of unsaturated fatty acids are related to the development of a rancid off-flavour. Lipid oxidation causes various quality problems such as rancidity, discolouration, shorter shelf-life, and increased health risks [43]; therefore, it must be prevented [3,32,44,45,46]. MDA (malondialdehyde) is very toxic secondary oxidation products, which are formed during oxidation, and its level needs to be controlled during the storage period both from a sensory point of view and the consumers’ health perspective [32,43]. In addition, MDA is widely used as a marker of meat oxidation [47], which is in good correlation with other meat oxidation indicators; for instance, the correlation coefficient between extracted TBARS and hexanal was 0.74, even in the case using coloured plant origin additives [48]. Therefore, it was selected in our study for evaluating the effects of additives. It should be noted that the measurement of TBARS provides preliminary information about oxidative processes in meat; for more sound support of antioxidative effects, the study should be extended using other methods such as measurement of peroxides, oxidation of meat pigments, and carbonyl and sulfhydryl groups.

**Figure 2:**
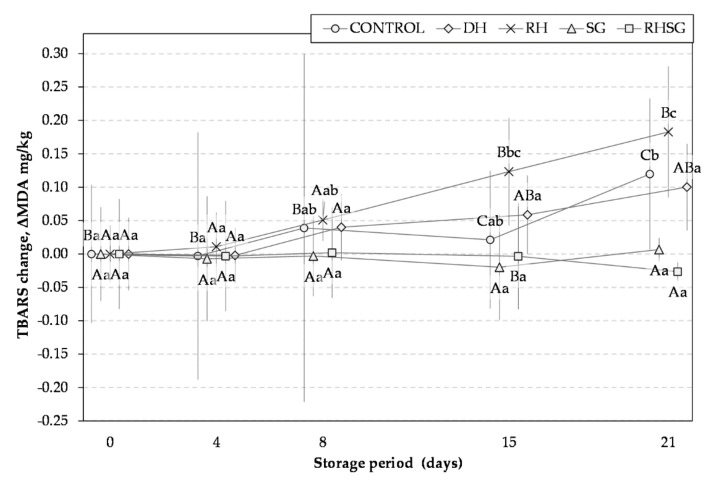
Changes in the TBARS values of grilled pork burger patties stored in the modified atmosphere during the storage period (ΔMDA mg/kg). Different capital letters express a significant difference between the variants within the same storage day by the Tukey’s test (*p* < 0.05). Different lower-case letters express a significant difference between the storage days within the same variant by the Tukey’s test (*p* < 0.05).

Thus far, as the method of measuring TBARS is based on visible light absorption, the initial values were conditionally equated to 0 and, afterwards, the changes were followed during storage (Figure 2). It is evident that the antioxidant SG extract fully stabilised the product in terms of the formation of TBARS during the whole period of storage. The TBARS values after 21 days in control samples increased from 0.420 to 0.540, while that in DH and RH samples increased from 0.197 to 0.297 and from 0.181 to 0.364, respectively.

The sample with RH after 15 and 21 days of storage reached the highest TBARS values, and it supports our hypothesis that unsaturated oil residues in the raw hemp seed press-cake may foster the formation of oxidation products. When SG was applied together with RH, the formation of MDA was fully inhibited: there were no significant changes in the TBARS values in the SG sample during the whole period of storage; it was in the range of 0.175 mg/MDA/kg. This finding supports our second hypothesis regarding the mitigation of oxidation processes in the case using raw hemp seed press-cake. In the case of using defatted hemp seed protein press-cake, the curve of formation of TBARS was almost similar to the control sample. It should be noted that endogenous lipophilic antioxidant vitamin E may strongly influence the oxidation process during storage or retail as it was recently reported by Smith et al. [49].

### 3.6. Effect of Additives on Sensory Attributes

Adverse effects on sensory quality are among the major problems in using morphologically different plant-origin ingredients in meat products. Therefore, the determination of acceptable doses of such ingredients and their effects on various organoleptic characteristics remain an important issue and challenge. Although nowadays the consumer’s preferences are also more strongly associated with the healthiness of plant-origin constituents, the sensory characteristics of foods have not lost any significance in determining their choice. Therefore, assessing the effects of the selected ingredients on the sensory quality of pork burger patties was among the most important tasks of this study.

**Figure 3:**
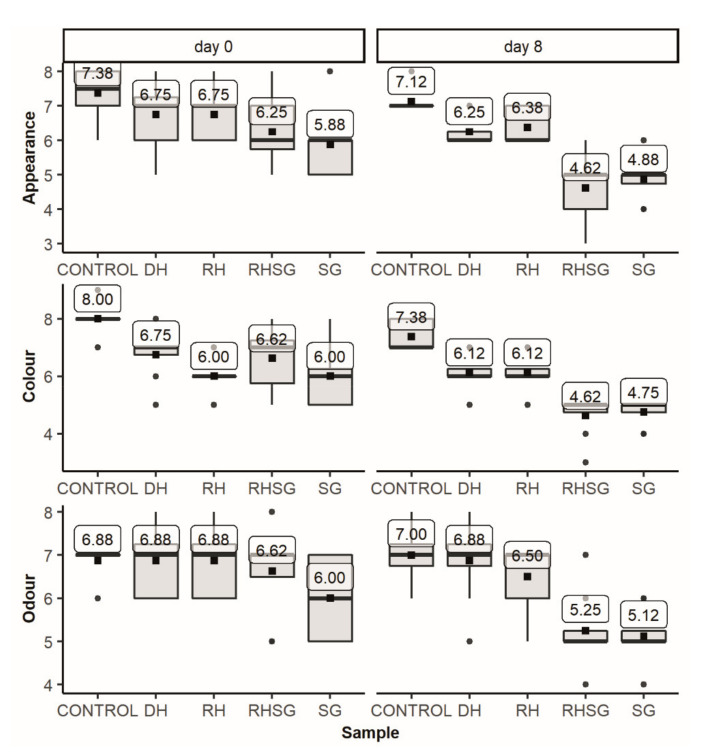
Sensory characteristics of raw pork patties with different plant-based ingredients on storage days 0 and 8 on the 9-point hedonic scale (control—without additives, RH—with 2% dried mechanically pressed hempseed cake, DH—with 2% defatted by supercritical CO_2_ extraction hempseed cake, RHSG—with 0.5% sweet grass extract and 1.5% dried pressed hemp seedcake, and SG—0.5% sweet grass extract).

**Figure 4:**
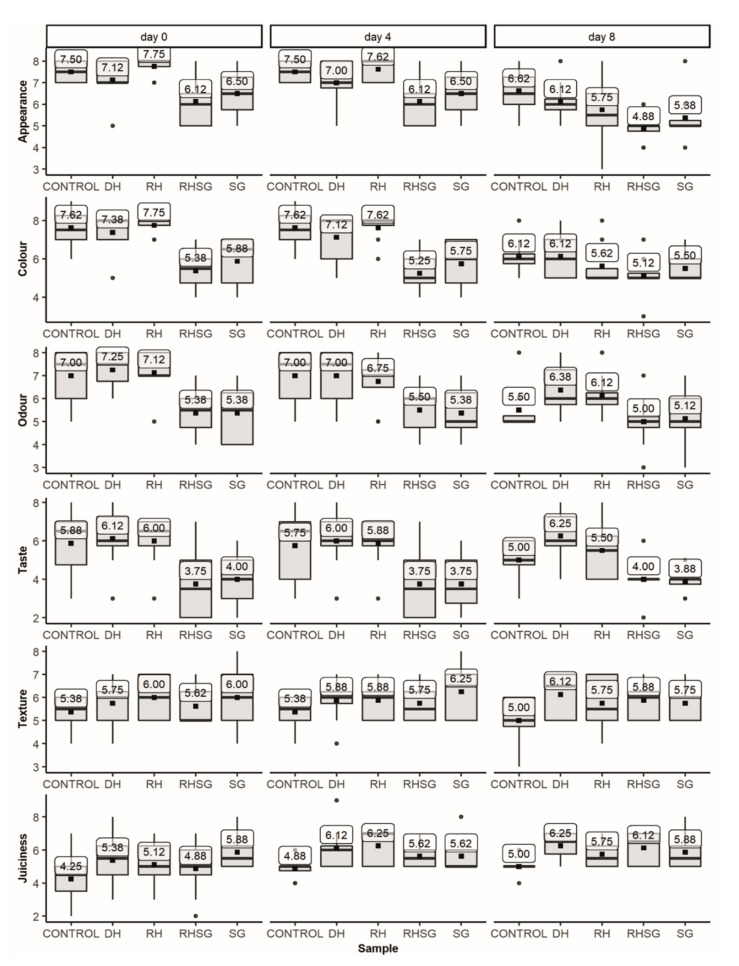
Sensory characteristics of grilled pork patties with different plant-based ingredients on storage days 0, 4, and 8 on the 9-point hedonic scale (control—without additives, RH—with 2% dried mechanically pressed hempseed cake, DH—with 2% defatted by supercritical CO_2_ extraction hempseed cake, RHSG—with 0.5% sweet grass extract and 1.5% dried pressed hemp seedcake, and SG—0.5% sweet grass extract).

For this purpose, the influence of the addition of plant-based ingredients on the sensory properties of raw and grilled pork patties was assessed, and the results are summarised in Figure 3 and Figure 4. The SG extract had the most notable effect on the sensory descriptors both in the case of raw and grilled patties. The panellists gave higher scores for the appearance and colour of the control sample, most likely, due to the lighter colour, which was also determined by the spectrophotometric method (Table 5); dark-green SG additives reduced the L* values by 16–20%. The score for the odour was above the acceptability or satisfactory limit (score = 5) for all raw patties.

Some changes were observed in the sensory evaluation scores during storage. For instance, the highest scores for appearance received raw patties without additives on days 0 and 8. The patties with DH and RH were evaluated with a similar range of scores for colour on days 0 and 8, while the most significant variations were observed for the patties with RHSG. The greatest variability of the odour scores was determined for freshly produced raw patties (day 0) with SG, while the control sample obtained more uniform assessments. In general, in the case of raw patties, the highest scores obtained control samples followed by the products with DH, RH, and SG additives.

In case of the grilled patties, the appearance was evaluated, with lower scores for the samples with RHSG 6.12 and 4.88 on days 0 and 8, respectively. However, the variability of the evaluation scores was observed for some sensory characteristics. It may be noted that the panellists pointed out greenish and therefore unusual meat product colour for the pork patties with SG. These patties also received lower scores for odour, e.g., on day 8, the average score was 5.12, while for the control sample, it was 7 (for the control, 100% of the results were between 6–8 and 50% of assessors gave the score 7, while for SG, the rating was even more uniform and 62.5% of the assessors gave the score 5). The scores for the patties with hemp seed additives were quite similar, from 6 to 7, for all assessed characteristics. It may be noted that the assessors for the grilled patties with DH and RH detected specific but generally acceptable nutty odour.

The grilled patties with SG received lower scores for all assessed parameters except the juiciness, when the evaluation was slightly higher than the products without additives. Comparing the evaluations during storage, the products with RHSG and SG received the most homogeneous rating for the appearance on day 8, while the results on days 0 and 4 varied more considerably. DH and RH ingredients also had positive effects on juiciness, while the taste of the patties with hemp seed press-cake also received good scores, on average 5.88 and 6.25, respectively. Consequently, quite high sensory evaluation scores for appearance, colour, and taste (>6 and low variability between the individual panellists) assigned to the patties with DH and RH indicate that hemp seed press-cake flour ingredients, in general, are acceptable for consumers. These findings should encourage the producers to apply promising and protein-rich hemp seed press-cake ingredients in the development of new meat formulas.

## 4. Conclusions

The results obtained confirmed both hypotheses raised for this study: (1) hemp seed press-cake ingredients that were not defatted increased the formation of oxidation products in meat patties and (2) the application of natural antioxidant extracted from sweet grass effectively inhibited the oxidation process, which was determined by measuring the content of malondialdehyde. Hemp seed press-cake added at 1.5–2.0% and sweet grass added at 0.5% had insignificant effects on the majority of the measured physicochemical characteristics of pork meat patties both after the addition and during storage, except for spectrophotometrically measured lightness L* value, which was significantly lower in the case using plant ingredients, particularly sweet grass extract. In addition, defatted hemp seed press-cake enabled the reduction in grilling losses to 14.34% (24.2 in control). In general, hemp seed press-cake ingredients did not have negative effects on the sensory characteristics of meat patties while the products with sweet grass extract were evaluated by the lower scores compared with other assessed samples. In conclusion, the results demonstrated that hemp seed press-cake ingredients may be successfully used in the production of pork meat burger patties, whereas the combination of the (raw) press-cake that was not defatted with sweet grass extract may substantially mitigate the pro-oxidative effects of residual and highly unsaturated hemp seed oil during storage. Further studies should focus on the possibilities of increasing the doses of hemp seed press-cake and on mitigating some negative effects of sweet grass on the selected sensory quality characteristics.

## Figures and Tables

**Table 1:** Antioxidant characteristics of sweet grass extract.

TPC (mg GA/g dw)	DPPH		ABTS^+^		ORAC, (mmol TE/g dw)
	(mg TE/g dw)	(IC_50_)	(mg TE/g dw)	(IC_50_)	
99.04 ± 1.61	300.2 ± 1.7	0.02	692 ± 8.2	0.09	30.65 ± 1.64

**Table 2:** Proximate composition of grilled pork patties and grilling losses. Values are least square means ± standard deviation.

Sample	Moisture (g/100 g)	Protein (g/100 g)	Fat (g/100 g)	Ash (g/100 g)	Grilling Loss (%)
Control	61.68 ± 4.94 ^a^	19.83 ± 1.61 ^a^	16.84 ± 6.53 ^ab^	2.48 ± 0.24 ^a^	24.20 ± 8.18 ^ab^
DH	63.36 ± 5.11 ^a^	18.84 ± 1.77 ^a^	15.85 ± 4.76 ^a^	2.59 ± 0.30 ^a^	14.34 ± 3.89 ^c^
RH	59.12 ± 4.30 ^b^	19.68 ± 1.29 ^a^	16.80 ± 5.80 ^ab^	2.59 ± 0.16 ^a^	20.89 ± 4.21 ^ad^
RHSG	58.82 ± 3.05 ^b^	18.82 ± 0.92 ^a^	16.68 ± 5.18 ^a^	2.45 ± 0.16 ^a^	19.45 ± 6.84 ^d^
SG	57.75 ± 2.94 ^b^	19.32 ± 0.86 ^a^	18.91 ± 4.20 ^b^	2.58 ± 0.25 ^a^	26.21 ± 5.91 ^b^

^a, b^^, c, d^ Different letters in columns indicate significant differences between means (*p* < 0.05) by Tukey’s multiple comparison’s post hoc test. Control—without additives, RH—with 2% dried mechanically pressed hempseed cake, DH—with 2% defatted by supercritical CO_2_ extraction hempseed cake, RHSG—with 0.5% sweet grass extract and 1.5% dried pressed hemp seedcake, and SG—0.5% sweet grass extract.

**Table 3:** Effects of additives and storage period (days) on the pH-value of grilled patties. Values are least square means ± standard deviation.

Sample	Storage Period (Days)				
	0	4	8	15	21
Control	6.08 ± 0.15 ^Aa^	6.09 ± 0.10 ^Aa^	6.13 ± 0.08 ^ABa^	6.11 ± 0.07 ^Aa^	6.15 ± 0.09 ^ABa^
DH	6.18 ± 0.09 ^BCa^	6.22 ± 0.16 ^Ba^	6.18 ± 0.10 ^Ba^	6.24 ± 0.11 ^BCa^	6.25 ± 0.12 ^Ca^
RH	6.19 ± 0.08 ^Cab^	6.21 ± 0.07 ^Babc^	6.18 ± 0.13 ^ABa^	6.29 ± 0.08 ^Cc^	6.27 ± 0.13 ^Cbc^
RHSG	6.18 ± 0.06 ^BCa^	6.16 ± 0.13 ^ABa^	6.17 ± 0.10 ^ABa^	6.20 ± 0.08 ^Ba^	6.20 ± 0.11 ^BCa^
SG	6.11 ± 0.07 ^ABa^	6.08 ± 0.08 ^Aa^	6.10 ± 0.08 ^Aa^	6.05 ± 0.09 ^Aa^	6.08 ± 0.09 ^Aa^

Least square means followed by the different capital letters in the columns and lower-case letters in the rows differ significantly by the Tukey’s multiple comparison’s post hoc test (*p* < 0.05). Control—without additives, RH—with 2% dried mechanically pressed hempseed cake, DH—with 2% defatted by supercritical CO_2_ extraction hempseed cake, RHSG—with 0.5% sweet grass extract and 1.5% dried pressed hemp seedcake, and SG—0.5% sweet grass extract.

**Table 4:** Effects of additives and storage period (days) on the aw-value of grilled patties. Values are least square means ± standard deviation.

Sample	Storage Period (Days)				
	0	4	8	15	21
Control	0.953 ± 0.022 ^Aa^	0.950 ± 0.022 ^Aa^	0.950 ± 0.021 ^Aa^	0.951 ± 0.021 ^Aa^	0.952 ± 0.016 ^Aa^
DH	0.957 ± 0.022 ^Aa^	0.953 ± 0.019 ^ABa^	0.956 ± 0.018 ^Ba^	0.957 ± 0.018 ^BCa^	0.955 ± 0.018 ^ABa^
RH	0.957 ± 0.017 ^Aa^	0.957 ± 0.018 ^Ba^	0.957 ± 0.019 ^Ba^	0.955 ± 0.021 ^ABa^	0.956 ± 0.017 ^ABa^
RHSG	0.963 ± 0.022 ^Ba^	0.963 ± 0.022 ^Ca^	0.964 ± 0.020 ^Ca^	0.961 ± 0.022 ^Ca^	0.963 ± 0.016 ^Ca^
SG	0.958 ± 0.020 ^Aa^	0.957 ± 0.022 ^Ba^	0.959 ± 0.021 ^Ba^	0.957 ± 0.021 ^BCa^	0.961 ± 0.017 ^BCa^

Least square means followed by the different capital letters in the columns and lower-case letters in the rows differ significantly by the Tukey’s multiple comparison’s post hoc test (*p* < 0.05). Control—without additives, RH—with 2% dried mechanically pressed hempseed cake, DH—with 2% defatted by supercritical CO_2_ extraction hempseed cake, RHSG—with 0.5% sweet grass extract and 1.5% dried pressed hemp seedcake, and SG—0.5% sweet grass extract.

**Table 5:** The changes in the colour parameters of pork patties during the storage period (days). Values are least square means ± standard deviation.

Sample	Storage Period (Days)				
	0	4	8	15	21
	Lightness L*				
Control	71.86 ± 7.89 ^Ca^	72.65 ± 8.16 ^Da^	72.79 ± 8.16 ^Da^	75.03 ± 6.39 ^Ca^	72.25 ± 7.82 ^Da^
DH	68.70 ± 9.19 ^BCa^	67.77 ± 11.66 ^BCa^	66.76 ± 11.01 ^BCa^	69.90 ± 8.91 ^Ba^	70.03 ± 8.03 ^CDa^
RH	67.70 ± 7.42 ^Ba^	69.20 ± 8.13 ^CDa^	68.10 ± 9.56 ^Ca^	69.26 ± 7.10 ^Ba^	67.60 ± 6.11 ^BCa^
RHSG	57.38 ± 8.81 ^Aa^	61.74 ± 6.36 ^Abc^	62.08 ± 7.94 ^Ac^	61.48 ± 6.79 ^Abc^	57.89 ± 8.67 ^Aab^
SG	60.50 ± 9.73 ^Aa^	64.39 ± 8.42 ^ABb^	63.67 ± 7.74 ^ABab^	64.63 ± 5.96 ^Ab^	64.18 ± 7.85 ^Bab^
	Redness a*				
Control	7.67 ± 5.45 ^Aa^	7.08 ± 4.38 ^ABa^	7.01 ± 4.45 ^ABa^	6.73 ± .59 ^ABa^	6.81 ± 4.54 ^Aa^
DH	7.62 ± 5.92 ^Aa^	7.57 ± 4.88 ^Ba^	8.01 ± 5.89 ^Ba^	7.83 ± 5.80 ^Ba^	7.29 ± 5.65 ^ABa^
RH	7.73 ± 6.02 ^Aa^	7.59 ± 6.29 ^Ba^	7.48 ± 6.04 ^ABa^	7.89 ± 6.29 ^Ba^	8.71 ± 6.49 ^Ba^
RHSG	6.55 ± 5.99 ^Aa^	6.07 ± 6.78 ^ABa^	6.28 ± 6.72 ^Aa^	6.41 ± 6.30 ^ABa^	6.33 ± 5.74 ^Aa^
SG	6.24 ± 6.45 ^Aa^	5.81 ± 6.29 ^Aa^	6.13 ± 6.56 ^Aa^	6.15 ± 6.80 ^Aa^	6.20 ± 6.08 ^Aa^
	Yellowness b*				
Control	28.06 ± 15.18 ^Aa^	27.51 ± 15.34 ^Aa^	28.07±15.24 ^Aa^	26.32±12.92 ^Aa^	26.54±13.29 ^Aa^
DH	28.40 ± 15.00 ^Aa^	28.48 ± 13.92 ^Aa^	28.86±14.14 ^Aa^	27.86±12.26 ^ABa^	25.93±11.91 ^Aa^
RH	30.26 ± 14.05 ^Aa^	29.29 ± 14.88 ^Aa^	29.73±14.92 ^Aa^	27.72±12.46 ^ABa^	26.74±12.40 ^Aa^
RHSG	36.06 ± 14.07 ^Bc^	33.13 ± 14.43 ^Babc^	34.30±14.57 ^Bbc^	31.05±11.46 ^BCab^	29.50±10.09 ^ABa^
SG	35.64 ± 14.23 ^Bb^	33.47 ± 14.65 ^Bab^	34.45±15.32 ^Bab^	32.23±11.71 ^Ca^	32.26±11.47 ^Bab^

Least square means followed by the different capital letters in the columns and lower-case letters in the rows differ significantly by the Tukey’s multiple comparison’s post hoc test (*p* < 0.05). Control—without additives, RH—with 2% dried mechanically pressed hempseed cake, DH—with 2% defatted by supercritical CO_2_ extraction hempseed cake, RHSG—with 0.5% sweetgrass extract and 1.5% dried pressed hemp seedcake, and SG—0.5% sweet grass extract.

## Data Availability

The data presented in this study are available from the corresponding author (A.T.) upon request.
